# Family Business Internationalization in Paradox: Effects of Socioemotional Wealth and Entrepreneurial Spirit

**DOI:** 10.3389/fpsyg.2021.667615

**Published:** 2021-04-22

**Authors:** Chenfei Jin, Bao Wu, Yingjie Hu

**Affiliations:** ^1^China Institute for Small and Medium Enterprises, Zhejiang University of Technology, Hangzhou, China; ^2^School of Management, Zhejiang University of Technology, Hangzhou, China

**Keywords:** socioemotional wealth, family reputation, family involvement, foreign investment, entrepreneurial spirit

## Abstract

This study investigates the internationalization (i. e., foreign investment) of small family businesses by classifying the effects of external socioemotional wealth (family reputation) vs. internal socioemotional wealth (family involvement). The study involved 2,704 small family businesses in China, and the results support the hypothesis that family reputation has a positive effect on internationalization, while family involvement has a negative effect on internationalization. Moreover, entrepreneurial spirit reinforces the positive effect of family reputation on internationalization and enhances the negative relationship between family involvement and internationalization. This study contributes by examining the effect of entrepreneurial spirit as a potential balancing factor for the paradoxical influence of internal vs. external socioemotional wealth.

## Introduction

To respond to the challenges brought about by economic globalization, internationalization strategies have become crucial or even necessary for many family businesses that traditionally operate in domestic markets. The organizational behaviors of family business differ from other types of firms with different ownership structures because family members usually intend to run the business by creating and preserving socioemotional wealth (SEW), even at the expense of financial gains (Chrisman et al., 2007; Chrisman and Patel, 2012). Studies have shown that family businesses are less likely to internationalize than non-family businesses. The risk-aversion of family businesses (Fernández and Nieto, 2005), deep local embeddedness (Gallo and Garcia Pont, 1996), concern for subsequent generations (Yang et al., 2018), and other family-related factors may result in different internationalization processes and internationalization strategies compared to non-family businesses. Hence, family business internationalization as a strategic decision is often affected simultaneously, and paradoxically, by professional rationale and family concerns about socioemotional wealth (Bell et al., 2004; George et al., 2005; Fernández and Nieto, 2006; Johanson and Vahlne, 2009).

Against such a backdrop, the importance of the socioemotional wealth of a family business on its internationalization is well-recognized. SEW is defined as the “affective endowment of family owners” (Gomez-Mejia et al., 2011), or “non-financial value accuring a family through its association with a firm” (Debicki et al., 2016). It is acknowledged that family businesses may pay particular attention to preserving and fostering SEW when they strategically consider internationalization. However, the specificities of family businesses make them heterogeneous in their SEWs, justifying the need to explore the effects of SEWs on internationalization in detail. To do so, this study adopts the research strategy of dimensionalizing the construct of SEW, exploring the effects of external SEW proxied by firm reputations and internal SEW proxied by family involvement in foreign investment of Chinese small family businesses. Family reputation based on the financial performance of the family business (Lange et al., 2011) and family involvement backed by family ownership (Bloom, 1985) are usually mentioned in exploring the black box of SEW and are regarded as valid and useful proxy variables in measuring SEW. This study considers how the internal and external dimensions of SEW are in tension and are associated with foreign investment in small family businesses.

This study explores the moderating effect of entrepreneurial spirit in foreign investment choices. Overall, this study theoretically and empirically advances current understanding of the internationalization of family businesses from a SEW perspective. In particular, the present study makes three unique contributions to extant literature about family business internationalization.

First, this study examines how the SEWs of small family businesses affect foreign investment by external vs. internal sources. Family heterogeneity should be carefully considered when we are talking about SEWs, and a good way of doing this is to detail the construct *per se* by dimensionalization. In most literature, the non-financial values associated with a family business are roughly labeled by SEW and indiscriminately treated and measured by proxies such as family ownership and family control on many occasions. In this study, we classify SEW into family reputation originating from the financial performance of the family business and family involvement backed by family ownership. The study thus explores the “black box” of SEW with empirical evidence in the context of foreign investment in Chinese small family businesses.

Second, we chose to investigate foreign investment, rather than export intensity or export propensity. Foreign investment is a deep-level internationalization process usually involving higher resource commitments. Studies have found that the SEW is heavily weighted in the family decision process when a heavy resource commitment is involved. Therefore, foreign investment offers an excellent context in which to explore the functions and effects of SEW on internationalization.

Finally, this study explores the moderating effect of entrepreneurial spirit on the relationship between SEWs and foreign investments. In previous studies, entrepreneurial spirit is usually missing from discussions about the SEW of a family business. Our study found that the priority ordering of SEW in a family business with a lasting entrepreneurial spirit or family business losing their entrepreneurial spirit is quite different. Therefore, as this study indicates, the effects of SEWs on foreign investment are distinct in these two contexts, contributing to perspectives on SEW in family business internationalization theory.

## Theory and Hypotheses

### Socioemotional Wealth and the Internationalization of Family Businesses

Following a pioneering study by Gallo and Sveen (1991), research about the internationalization of family businesses has steadily been the subject of growing interest from scholars from multiple disciplines (Kontinen and Ojala, 2010; Pukall and Calabrò, 2014; Arregle et al., 2016). The majority of literature on family firm internationalization focuses on the relationship between family ownership and internationalization (Pukall and Calabrò, 2014; Arregle et al., 2016; Dou et al., 2020). Though there are strong arguments that family business may be reluctant to internationalize for the intention of preserving Socioemotional Wealth (SEW), existing empirical studies offer contradictory predictions about either positive (Minetti et al., 2015; Fang et al., 2018), negative (Sanchez-Bueno and Usero, 2014; Alessandri et al., 2018), curvilinear (Liang et al., 2014), or no relationship (Cerrato and Piva, 2012) between internationalization and family ownership. Family-specific heterogeneity is an important factor in the strategic process of internationalization of family businesses (Pukall and Calabrò, 2014; Yang et al., 2018).

In past years, perspective of socioemotional wealth has attracted academic interest as an insightful approach for explaining why, when, and how “familiness,” meaning family-specific factors (Habbershon et al., 2003), or the affect-related non-financial values of families exert influence on the internationalization of family firms (Yang et al., 2018; Dou et al., 2020). It is acknowledged that family businesses would pay more attention to the balance between financial performance and socioemotional value than non-family businesses. Long-term orientation and shared values for the future are important features of a family business, which are more or less related to family ownership and family control of the business (Kotlar and de Massis, 2013). Therefore, the psychology-driven motives of a family business for internationalization (Schulze and Kellermanns, 2015) defers from non-family business, family-specific characteristics, and SEWs and is likely to shape their values, missions, and objectives (Jaskiewicz et al., 2017). This intention to preserve SEW is associated with conservative strategy and risk aversion in the context of a family business (Zahra, 2005). These types of businesses tend to internationalize later, more slowly, and more prefer to internationalize in geographically or culturally similar countries (Claver et al., 2007; Graves and Thomas, 2008; Jiang et al., 2020; Xu et al., 2020). Furthermore, family businesses are also assumed to follow the stage model, preferring to lower resource commitments (Pukall and Calabrò, 2014) in their internationalization process. SEW perspective offers more rich explanations about the organizational modes of expanding internationally in the context of a family business.

It has been widely acknowledged that the primary reference point of a family business in making strategic decisions is not only economic tradeoff but also SEW preservation (Gómez-Mejía et al., 2007; Berrone et al., 2012; Jiang et al., 2020). Although the SEW approach has been widely used to explain unique considerations about socio-affective utilities in family strategic decisions, the heterogeneity of SEW ordering among family businesses has not been explored in detail. It is necessary to shed light on the conceptual nature of SEW, which is roughly defined as the affective endowment or non-economic, affective values that a family derives from its ownership in the family business (Gómez-Mejía et al., 2007; Gomez-Mejia et al., 2010, 2011; Berrone et al., 2012). In previous studies, the percentage of family ownership has been usually employed as a general operational measure for SEW. Although family ownership theoretically leads to affective values for the owning family, this simplified indicator is not sufficient to express the various multifaceted aspects of SEW. It is, therefore, necessary to explore the multidimensionality of SEW to match heterogenetic priority ordering of the owning family when they define their SEW pursuits. Various effective utilities, including the perpetuation of a positive family image and reputation, the need for identification, a sense of belonging, enjoyment of personal control, the ability to exercise authority, and an active role in the family dynasty, have been mentioned as the content of SEW (e.g., Gomez-Mejia et al., 2010; Berrone et al., 2012; Debellis et al., 2020; Jiang et al., 2020). It is suggested that families may vary their preference for internal and external sources of SEW, depending on family context (Debicki et al., 2016). For example, even though the first inclination of the owning family is to enhance family ownership to preserve SEW, family concern for SEW may dynamically shift toward family reputation if the external sources of SEW do not enhance family ownership (Vardaman and Gondo, 2014). The external and internal sources of SEW may be associated with organizational behaviors in differential ways (Miller and le Breton-Miller, 2014). For example, an owning family emphasizing the firm's reputation is likely to place high importance on business performance, while another family concerned about increased family involvement may not (Naldi et al., 2013; Debicki et al., 2016). This study focuses on two typical external and internal sources for SEW: family reputation originating from the financial performance of a family business and family involvement backed by family control over the business. Furthermore, the heterogenetic priority of SEW pursuits is dynamically changed and may be associated with a family-specific context.

This study discusses foreign investments in Chinese family businesses. Foreign investment, formally called Outward Foreign Direct Investment (OFDI), is a typical mode of internationalization with heavy resource commitment (Kao and Kuo, 2017; Yang et al., 2018). When heavy resource commitment involves disputes about internationalization among family members will be amplified (Jiang et al., 2020; Xu et al., 2020). The potential threat of internationalization to SEW will loom salient in such cases. The purpose of foreign investment by Chinese small family business explored in this study is expansion, which is related to foreign investment targeted to industrial sectors. Family ownership provides the foundation for the intention to preserve SEW endowment to build a family legacy (Zellweger et al., 2007). The attitude of the owning family to foreign investment will shape their pursuit of SEW in different contexts.

**Figure 1:**
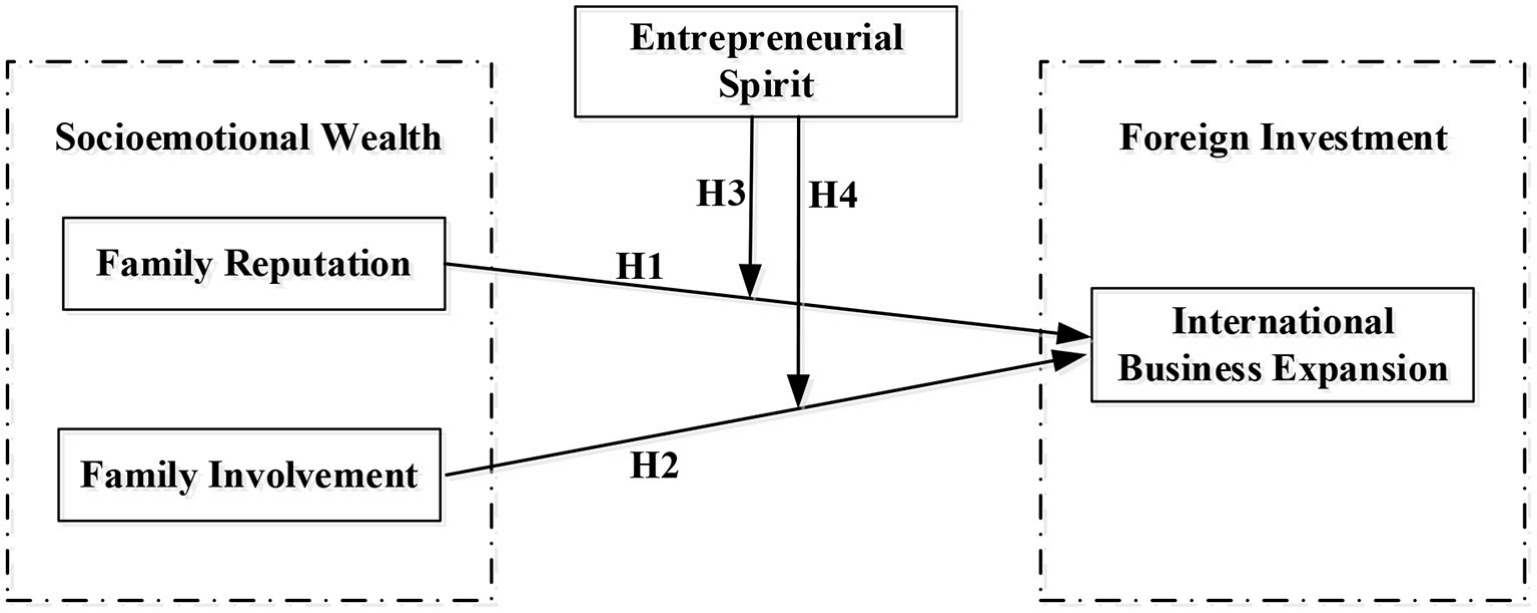
Conception model.

The international business expansion involves entrepreneurship for most owning families (Oviatt and McDougall, 2005; Yang et al., 2018). Usually, the family business will face a resource gap in the internationalization process and have to seek capital, skill, and capabilities from external resources, stakeholders, and institutions (Jiang et al., 2020; Xu et al., 2020). International expansion implies changes in strategies and organizational structure to meet the demands of entrepreneurship activities is full of uncertainty in a foreign country. Prior studies have proposed that family members may be suspicious of structural changes or afraid of losing family influence (Mitter et al., 2014; Chua et al., 2018). Aversion to the loss of SEW would restrain foreign investment decisions and exert influences on intention and mode of international expansion. There is also another argument that international expansion is a reputational signal for a family business in emerging markets. The aspiration to develop SEW endowment may inspire the owning family to engage in international expansion in some family contexts (Gomez-Mejia et al., 2010). The attitude and intention of foreign investment are largely moderated by the entrepreneurship of the owning family. The priority SEWs of the owning family with a strong entrepreneurial spirit would differ from that of an owning family with a weak entrepreneurial spirit. Therefore, the entrepreneurial spirit of the owning family is an important moderating variable in the strategic process, especially when the first generation of the Chinese owning family is facing the problem of succession. See Figure 1 for conception model.

### Family Reputation and Foreign Investment

A distinction between external (e.g., family reputation) and internal (e.g., family involvement) sources for SEW has been explicitly or implicitly proposed in the literature on this subject (Block, 2010). Family reputation is usually perceived as an important external source for SEW (Gomez-Mejia et al., 2010; Berrone et al., 2012; Naldi et al., 2013). The family business places high importance on family reputation and firm image over short-term financial benefits (Gomez-Mejia et al., 2011). This great concern for family reputation makes the family business more sensitive to the potential threat of negative images caused by business strategy. The family business has a stronger preference for a positive reputation (Dyer and Whetten, 2006), for instance, the inclusion of the family surname in the business name is positively associated with the family business' social responsibility (Uhlaner et al., 2004).

In other aspects, reputation building offers motivation for the owning family to seek business success. A firm's reputation is defined as the “beliefs of various stakeholders regarding the likelihood that the firm will deliver value along key dimensions of performance (Rindova and Fombrun, 1999), chiefly product quality and financial performance” (Rindova et al., 2006). Therefore, reputation serves as “a signal of future performance based on perceptions of past performance” (Dimov et al., 2007, p. 486). Potential business partners may choose to cooperate with the family business because of its reputation and the fact that it is run by a trustworthy family. Successful international business expansion is a strong signal for better performance and can create a more positive image for the firm in emerging markets. For example, international product brands or local firms with successful outward foreign direct investments are usually overvalued in the Chinese market.

The Chinese government also encourages firms to invest abroad, and the volume of Chinese OFDI has been increasing in recent years (Yang et al., 2018). Family businesses with foreign investment are usually more appreciated by local governments (Jiang et al., 2020; Xu et al., 2020). In such a context, successful expansion in international markets will bring a more positive reputation to the owning family and offer more advantages in establishing and maintaining a good relationship with local governments (Xu et al., 2020). In this way, reputation-building offers more motives to expand the business internationally. This study thus proposes the following hypothesis:

*Hypothesis 1: there is a positive relationship between family reputation and international business expansion*.

### Family Involvement and International Business Expansion

Family involvement is usually proposed as a core aspect of internal sources for SEW (Gómez-Mejía et al., 2007; Cabeza-García et al., 2017). The effect of family involvement on entry mode and the geographical choice of foreign investment is focused at the intersection of the family business and international business disciplines. For example, Liang et al. (2014) propose that family businesses with a high level of family involvement will assign a higher priority to risk-aversion to SEW than those with lower family involvement (Choi et al., 2015).

The strategic decision to undertake internationalization may present a salient threat to SEW, especially when heavy resource commitment is involved (Gomez-Mejia et al., 2010). Foreign investment usually requires more international human resources, knowledge, skills, and funding from outside sources (Fatemi, 1984), as these skills and resources may not be available within the family (Schulze et al., 2003; Hitt et al., 2006). To acquire such external resources, the internationalization process may coincide with the loss of family control over their business, which most family businesses want to avoid (Koropp et al., 2014). Furthermore, a family business might be pressured to change its business strategy and corporate governance to meet the demands of the family's international business expansion. This could increase external professionals, outside managers, investment partners, and other stakeholders involved in the process of foreign investment. The involvement of family members may thus decrease and lead to a loss of control over firm affairs and decision-making power (Berrone et al., 2012; Zellweger et al., 2012). In the majority of studies on SEW, family control and the involvement of family members in the firm's business are the core internal sources for SEW. Maintaining family involvement and influence over the firm usually gain higher priority than risk diversification advantage or entrepreneurial opportunity of international business expansion (Yang et al., 2018). Thus, considering the potential loss of SEW related to foreign investment in other countries, a family business with greater family involvement is less likely to initialize a foreign investment strategy.

The social embeddedness of family members offers more physiological reasons for the owning family to resist strategic investment abroad (Chua et al., 2018). Most of the Chinese family businesses rise along with the opening and reform of Chinese governance and society. The social ties and emotional connections of family members with local communities are regionally bounded (Banalieva and Eddleston, 2011; Jiang et al., 2020). For most Chinese owning families, social capital that is also regionally bounded offers important advantages when operating a family business (Wu, 2018). Family businesses with greater family involvement are likely to focus their investment on familiar domestic markets. Accordingly, this study proposes the following:

*Hypothesis 2: there is a negative relationship between family involvement and international business expansion*.

### The Moderating Effects of Entrepreneurial Spirit

The priority ordering of SEWs is family-specific and context-specific. The conceptual nature of SEW grasps the diversity and valence of affective values derived from family control (Miller and le Breton-Miller, 2014; Vardaman and Gondo, 2014; Chua et al., 2015; Schulze and Kellermanns, 2015). The meaning of this concept is so rich that it is expressed in terms of its multidimensionality, and accordingly, this study focuses on the context-specific dynamics of the priority ordering of SEWs.

Entrepreneurial spirit offers an insightful perspective for the dynamic priority ordering of SEWs. The entrepreneurial spirit in our study is close to an entrepreneurial orientation defined as “behavior of the business characterized by innovation, proactivity and risk-taking” (Miller, 1983): and conceptualized as the capacity of the firm to undertake activities related to innovation, assumption of risk and pioneering new actions (Engelen et al., 2015). Any attempt at international business expansion is essentially entrepreneurial as it represents a combination of risk-taking, innovation, and proactiveness (Javalgi and Todd, 2011). The concept of entrepreneurial spirit is measured as an inclination for being entrepreneurial, innovative, and risk-taking in the context of Chinese economic transformation in our study shapes the strategic thinking of the owing family about the priority ordering of SEWs (Memili et al., 2020). This argument is supported by literature about how SEW interplay with psychological capital (Memili et al., 2020; Tsai et al., 2020). Small family businesses with a strong entrepreneurial spirit are expected to place higher priority on family reputation and the lasting prosperity of the family business, and those with a low entrepreneurial spirit are expected to highly appreciate risk-aversion and maintain family control. Particularly, when the first generation of small family businesses is facing the challenge of succession, entrepreneurial spirit increasingly gains significance in determining SEW priority order. A failure in transgenerational entrepreneurship will exacerbate the decline of entrepreneurial orientation (Jaskiewicz et al., 2015). This study attempts to link the family ordering of SEWs to the moderation of entrepreneurial spirit, particularly the impact of entrepreneurial spirit on the priority shift between the external and internal sources of SEW, and its influence on the investment modes of a family business in foreign countries.

The attitude and strategic choice of foreign investment in a family business with a strong entrepreneurial spirit is expected to differ from that in a family business with a weak entrepreneurial spirit. Previous studies confirm entrepreneurial orientation positively influences the international performance of the family business (Hernandez-Perlines, 2018). This perspective of entrepreneurial orientation is used as a dynamic way of explaining why companies become internationalized (Freeman and Cavusgil, 2007; Sundqvist et al., 2012). Our study argues that the presentence or absence of entrepreneurial spirit will moderate the relationship of multiple dimensions of SEW and international business expansion.

The perspective of entrepreneurial orientation addresses how entrepreneurial spirit stimulates decision-making through the search and exploitation of opportunities in a proactive, innovative, and risky way (Hernandez-Perlines, 2018). Benefiting from a strong entrepreneurial spirit, the family business is more likely to expand its SEW along with their entrepreneurial expansion of international business (Yang et al., 2018). International business expansion is a good signal reflecting the strength, capability, and prestige of family business in China's domestic market. Successful international business expansion is good for improving the reputation and image of a family business. It is expected that these family businesses with a strong entrepreneurial spirit place greater importance on family reputation based on business performance and a positive attitude to international business expansion (Javalgi and Todd, 2011). Conversely, the absence of entrepreneurial spirit usually leads to risk-aversion strategy and the owning family may place more importance on saving the current stock of SEW and stress on maintaining family control and influence on the family business. In this instance, reputation-building motives for international business expansion are necessarily weakened. A family business with a low entrepreneurial spirit usually intends to liquidate its assets rather than initialize international business projects. These family businesses would like to preserve wealth for the owning family in a risk-averse way, such as maintaining the current domestic market share rather than start a risky international entrepreneurial activity. Reputation-building based on international business expansion loss priority in SEW is by order of the owning family. Thus, the connection between family reputation and family business expansion is relatively strong due to entrepreneurial spirit. Accordingly, this study proposes the following:

*Hypothesis 3: the presence of entrepreneurial spirit will positively moderate the relationship between family reputation and international business expansion*.

It is a reasonable assumption that entrepreneurial spirit is positively associated with a positive attitude to change, outside resources, professionals and managers, and aggressive business strategy, etc. With a strong entrepreneurial spirit, the family business usually is more willing to push forward business expansion at the cost of decreasing the extent of family involvement. These family businesses usually weigh financial goals heavily and to some extent refrain from the negative effect of family involvement on international business expansion. Such family businesses with a strong entrepreneurial spirit usually place their priority on creating SEW along with international business expansion and are optimistic about the potential loss of SEW caused by decreasing family involvement. The presentence of a strong entrepreneurial spirit may enable them to release this perception of the links between family involvement and risk-aversion. The social embeddedness that is tightly associated with family involvement is not likely to be changed by a strong entrepreneurial spirit. The combination of these traits of family involvement and entrepreneurial spirit in the owning family is more likely to facilitate the family business to expand in the domestic market, especially considering that the Chinese domestic market is one of the fastest-growing in the world. Focus on the domestic market is a reasonable strategic choice for family businesses with a higher level of family involvement and entrepreneurial spirit. Putting a higher priority on domestic expansion means a declining likelihood of them initiating international business expansion.

The picture in a family business with a weak entrepreneurial spirit is expected to be quite different. Weak entrepreneurial spirit means less likeliness of actual business expansion both in domestic markets and international markets. The absence of entrepreneurial spirit may enhance the inclination of risk-aversion that is closely linked with a high level of family involvement. If there is a lack of entrepreneurial spirit, these family businesses like to preserve wealth for the owning family in a risk-averse way. Besides, there may be concerns about the intra-generational transfer of family wealth. To some extent, abroad financial investment offers a feasible way to preserve business capital by diversifying its international wealth allocation. International business expansion, in some special but not unpopular cases, offers a suitable way to transfer family wealth internationally. The absence of entrepreneurial spirit may relieve the negative effect of family involvement in international business expansion. Then, the absence of entrepreneurial spirit should offset part of the negative impact of family willingness for international business expansion. Thus, the negative connection between family involvement and international business expansion in a family business with strong family involvement is relatively stronger than that in a family business with weak family involvement. Accordingly, this study proposes the following:

*Hypothesis 4: the presence of entrepreneurial spirit will reinforce the relationship between family involvement and international business expansion*.

## Materials and Methods

### Data

The data used in this study are based on the 12th China Private Enterprise survey (CPES) in 2016, which is a nationwide aerial survey on Chinese private entrepreneurs jointly conducted by the All-China Federation of Industry and Commerce (ACFIC), State Administration for Industry and Commerce (SAIC) and Chinese Academy of Social Science (CASS) since the beginning of the 1990s. Each wave of the survey covers about 0.055% of private firms in China mainland and 31 provincial regions, including 22 provinces, four municipalities directly under the supervision of the central government, and five minority autonomous regions.

To achieve a balanced representation across all regions and industries in China, a multistage-stratified random sampling method was used in this survey. The sampling procedure was conducted as follows: in the first step, we determined the total number of private enterprises surveyed. This total national sample size was assigned to 31 provinces in mainland China according to their shares of local private enterprises in the national total. Then six cities or counties were picked up for each province, generally including the provincial capital city, one prefecture-level city, one county-level city, and three counties. Then the number of private enterprises surveyed in each city/county and industry are in turn likewise determined according to its share of private number in this province or industry. Finally, private enterprises were randomly selected for each sub-sample. This dataset is by far the best for studying research issues concerning Chinese private enterprises because of its large sample cover. This survey is based on detailed household interviews with the majority owner of each selected private firm (Gao and Hafsi, 2015). The face-to-face data gathering process largely ensured the availability and accuracy of sensitive information on private enterprises, including family conception, internationalization, and personal characteristics, etc. It is acknowledged that this dataset is widely used for studying private entrepreneurs, family business, and other related topics.

The majority of private firms in China are under family ownership and are small businesses. In the 12th China Private Enterprise survey, the average size of sampled enterprises included ~215 employees, and the annual revenue of these enterprises was about 140.19 million RMB. The average share of family ownership was 79.9%, and about 58.4% of the sampled firms are held by an owning family, with 95.8% of sampled firms controlled by family ownership. It is well-accepted that the agency problem of corporate management is not a prominent issue in these family-owned small businesses (Du et al., 2015). Hence, our focus on family-controlled small businesses allows us to intensively explore the effect of SEWs on foreign investment in a family business by controlling the agency problem. The 12th China Private Enterprise survey contains 8,111 initial observations. After deleting those observations with missing data, this study obtained the final sample, including 2,704 observations. Please see Table 1 for details of the sample descriptions.

**Table 1:** Description of sampled companies.

**Region**	**Samples**	**Industry**	**Samples**
Eastern	1,535	Non-manufacturing	1,674
Central	674	Manufacturing	1,030
Western	495		
Total	2,704	Total	2,704
**Family ownership**	**Samples**	**Firm size (million)**	**Samples**
0–25%	257	0–10	1,211
25–50%	271	10–50	614
50–99%	600	50–100	291
100%	1,576	>100	588
Total	2,704	Total	2,704

### Measurement of International Business Expansion

International business expansion is usually measured by export intensity (Bausch and Krist, 2007; Elango and Pattnaik, 2007), export propensity (Ganotakis and Love, 2012; Yang et al., 2018), OFDI intensity (Vermeulen and Barkema, 2002; Bhaumik et al., 2010; Chari, 2013), and OFDI propensity (Hu and Cui, 2014; Liang et al., 2014). In this study, we focus on foreign investment by Chinese family-controlled small businesses. Following previous studies (Liang et al., 2014; Haapanen and Tapio, 2016; Jiang and Holburn, 2018; Yang et al., 2018), we constructed a dummy measurement for international business expansion depending on whether the family business is engaged in real foreign investment. Family business owners were asked to report the volume of their foreign investment in 2015 and illustrate the investment destination of their foreign investments in this survey. The classification of the usage destination of foreign investments in the surveys is as follows: (1) building overseas plants; (2) establishing overseas marketing branches; (3) merge, acquisition or investment in a foreign enterprise; (4) investing in real estate property in foreign countries; (5) purchasing natural resources, energy resources, and land overseas; (6) establishing oversea research and development branches; (7) investment immigration for the business owner or family members; and (8) others.

International business expansion was the main purpose of foreign investment in this study. The dummy variable of international business expansion was constructed by valuing it equal to 1 if one of the investment destinations is (1), (2), (3), (5), (6), or (8), otherwise equal to 0. Referring to sample statistics, we found that only 9.69 and 5.27% of sampled firms have Foreign Investment and international business expansion, respectively. In the CPES survey, the respondents reported the amount of OFDI. All of the family businesses that self-reported the investment destinations also reported the investment amount, providing an additional validity check of this dummy measurement.

### Measurement of Family Reputation and Family Involvement

The conceptualization of organizational/family reputation “consists of familiarity with the organization, belief about what to expect from the organization in the future, and impressions about the organization's favourability” (Lange et al., 2011, p. 153). The political status of a family business offers a strong signal about its reputation because official political titles can easily improve the visibility of a family business among business social networks, enhance the impression of business image and increase the favorability of the family business. Research by Du et al. (2015) supports the argument that political connection directly contributes to a firm's reputation. Den Hond et al. (2014) argues that corporate political activities affect firm reputation and that gaining access to and the attention of politicians contributes to positive reputation. The “corontion” of political title is an official recognition of the social reputation of a family business in the Chinese political context. Hence, this study measures family reputation by their political positions in the political system of the Chinese People' Congress (CPC) or Chinese People's Political Consultative Conference (CPPCC), and their position in the Local Federation of Industry and Commerce (LFIC). An ordinal variable is constructed based on the highest rank of CPC, CPPCC and LFIC the family business owner serves (Not titled = 0, County level = 1, Prefectural level = 2, Provincial level = 3, and National level = 4). After referring to the sample statistics, we found that 21.01, 29.12, and 65.27%, respectively, of sampled family business owners, serve or served in the CPC, CPPCC, and LFIC system, and that 61.1% of family business owners self-reporting for LFIC also occupied a position in CPC or CCPCC.

The literature on this subject observes that “components of involvement” and “essence” approaches are often used to measure family involvement in the family firm (Chrisman et al., 2005). The involvement approach focuses on the owning family's involvement in ownership, management, or control (Chrisman et al., 2005; Zellweger et al., 2010), whereas the essence approach focuses on “behaviors that produce distinctiveness before the firm can be classified a family firm” (Pearson et al., 2008, p. 966).

Involvement proxied by family ownership and management control is not sensitive in this study, since family ownership is generally high among all the sampled firms. In the survey, the value of family involvement is measured using the following items: (1) The owning family should retain over 50% ownership of the family firm; (2) Strategic decisions about the family business should be made by family members; and (3) The key positions should be occupied by family members. All items are measured using a five-point Likert scale anchored between “strongly disagree” and “strongly agree.” The internal consistency of the family involvement scale is measured using Cronbach's alpha. The three items are highly correlated (Cronbach's coefficient alpha = 0.82), confirming that the measuring scale is suitable for the present purposes. The Kaiser–Meyer–Olkin (KMO) measure of sampling adequacy value is 0.68, greater than the recommended level of 0.6. Bartlett's test of sphericity is also statistically significant (*p* < 0.000). These results suggest that it was appropriate to proceed to the factor analysis. One factor was subsequently extracted via principal component analysis with varimax rotation using the Kaiser normalization rotation method, and the total variance explained by this factor was 73.5%. Thus, the three items were averaged into an operating measure of family involvement.

### Measurement of Entrepreneurial Spirit

An entrepreneurial spirit is a value that enables an entrepreneur to look beyond accepted boundaries and find innovative ways to leverage the business and improve its practices. In the context of Chinese economic transformation and industrial upgrading, the entrepreneurial spirit is embodied in the pursuit of product innovation, technological innovation, and strategic transformation. Our measurement of entrepreneurial spirit is close to Miller's definition of entrepreneurial orientation as the “behavior of the business characterized by innovation, proactivity and risk-taking” (Miller, 1983: 771). Entrepreneurial orientation can also be defined as the capacity of a firm to undertake activities related to innovation, assumption of risk, and pioneering new action (Engelen et al., 2015). For companies struggling for survival in the transformation process of the Chinese economy, innovation must be addressed. In this study, entrepreneurial spirit is measured using the following items: (1) compared to your rivals, does your company have competitive advantages in core R&D team, core technologies, or technological talent? (2) Compared to your rivals, has your company invested in new product development? (3) In adapting to dynamic environmental changes, is your company willing to invest intensively in risky upgrading of products, reducing pollution, or diversification through technological innovation and product innovation? Total scores are obtained by assigning 1 for a positive answer and 0 otherwise. This measurement was constructed from existing official questionnaires, which differ from traditional scales for entrepreneurial spirit and entrepreneurial orientation (e.g., Covin and Slevin, 1989; Brown et al., 2001). In our sample, the KMO measure of sampling adequacy value is 0.64, above the recommended level of 0.6. Bartlett's test of sphericity is also statistically significant (*p* < 0.00). The internal consistency of the entrepreneurial spirit scale is measured using Cronbach's alpha; the three items are highly correlated (Cronbach's coefficient alpha = 0.70). These results suggest that the scale for entrepreneurial spirit used in this study is relatively reliable.

### Other Control Variables

This study uses a number of control variables for firm characteristics. Previous research has suggested that foreign investment is essentially capital flight across borders in an attempt to flee a worsening business environment, and it is reasonable to assume that the domestic business environment is a key factor influencing foreign investment decisions. In the survey, the domestic business environment is evaluated using 14 items covering administrative approval, honesty, and efficiency of public officials, fair enforcement of laws, intellectual property protection, personal security, protection of property, infrastructure, business service in the market, interference from local government, financing from banks and private sources, and availability of skilled workers. A factor extracted from these 14 items is used to control for the family business owner's perception of the domestic environment. This study assumes that in firm-level investment strategy, domestic investment is closely linked to foreign investment. International diversification is a strategic consideration within a firm's diversification strategy and may be connected with trends in industry diversification. Therefore, domestic investment and industry diversity are controlled. Education background is also controlled for using dummy variables for high school (or equivalent) and college degree and above, with junior high school and below as the default educational background.

Larger family firms may exhibit a greater inclination toward internationalization (Acedo and Casillas, 2005), and family influence and control over the family business, along with SEW structure, may vary with firm size (Gomez-Mejia et al., 2011; Pukall and Calabrò, 2014). Accordingly, this study controls for firm size, measured as the logarithm of the number of employees (Cesinger et al., 2016). Outward foreign investment is closely connected to financial status in terms of total assets and profitability. Consistent with the literature, this study adopts the logarithm of profit and net assets as control variables. The industry effect of internationalization, particularly between the manufacturing sector and other sectors, has often been mentioned in previous studies (e.g., Carpenter and Fredrickson, 2001; Yang et al., 2018). and this study uses a dummy variable to control for the industry effect of manufacturing. Regional heterogeneity is treated in line with the literature, with dummy variables constructed to control for regional effects of central and western China, taking eastern China as the default. In analyses not reported here, this study uses more fine-grained controls for industry and region; these do not affect the results and are omitted here for parsimony (Bernerth and Aguinis, 2016; Yang et al., 2018).

## Results

As international business expansion (IBE) is a binary dependent variable, this study applies a Probit model to test the hypotheses (e.g., Fernández and Nieto, 2005). The descriptive statistics and correlations for the variables are shown in Table 2. IBE is correlated with family reputation, family involvement, entrepreneurial spirit, and other control variables, demonstrating that these are empirically connected. The correlations between independent variables are moderate, demonstrating that they are empirically distinct.

**Table 2:** Descriptive statistics and correlation.

	***N***	**Mean**	**Sd**	**1**	**2**	**3**	**4**	**5**	**6**	**7**	**8**	**9**	**10**	**11**	**12**	**13**
IBE	2,704	0.05	0.22	1												
Family reputation	2,704	0.55	0.57	0.12^*^	1											
Family involvement	2,704	3.38	1.16	−0.13^*^	−0.12^*^	1										
Entrepreneurial spirit	2,704	1.13	0.83	0.15^*^	0.18^*^	−0.15^*^	1									
Profit	2,704	3.89	2.68	0.12^*^	0.41^*^	−0.05^*^	0.27^*^	1								
Equity	2,704	7.04	2.56	0.08^*^	0.41^*^	−0.08^*^	0.24^*^	0.47^*^	1							
Business environment	2,704	3.80	0.63	0	−0.11^*^	0.05^*^	0	−0.03	−0.06^*^	1						
Domestic investment	2,704	3.92	3.05	0.13^*^	0.41^*^	−0.13^*^	0.33^*^	0.52^*^	0.47^*^	−0.07^*^	1					
Diversification	2,704	0.28	0.45	0.03	0.17^*^	−0.10^*^	0.02	0.12^*^	0.12^*^	−0.11^*^	0.16^*^	1				
Firm size	2,704	3.91	1.80	0.11^*^	0.55^*^	−0.14^*^	0.31^*^	0.62^*^	0.58^*^	−0.09^*^	0.60^*^	0.17^*^	1			
Edu	2,704	2.00	0.45	0.05^*^	0.20^*^	−0.10^*^	0.13^*^	0.15^*^	0.14^*^	0.02	0.14^*^	0.06^*^	0.17^*^	1		
Region	2,704	1.62	0.78	−0.05^*^	−0.03	−0.07^*^	−0.09^*^	−0.08^*^	−0.16^*^	−0.07^*^	0	0.10^*^	−0.12^*^	−0.06^*^	1	
Manu	2,704	0.38	0.49	0.05^*^	0.13^*^	0.01	0.27^*^	0.22^*^	0.35^*^	0.03	0.22^*^	−0.16^*^	0.34^*^	0.02	−0.28^*^	1

*IBE, International business expansion*.

* *p < 0.05*.

Table 3 shows the results of all the hypothesis tests, based on the Probit regression. Bootstrapping is used to generate standard errors and t statistics. Model 1 includes the control variables, showing that entrepreneurial spirit (coefficient = 0.25, *p* < 0.01), domestic investment (coefficient = 0.07, *p* < 0.01) and profit (coefficient = 0.04, *p* < 0.05) have positive significant effects on the international expansion of a family business, while business environment and firm size have no significant effects. Slack resources offered by profitability may be important in the implementation of internationalization strategies (Yang et al., 2018), including IBE. The results from Model 1 indicate that IBE (i.e., industrial investment abroad) is not significantly associated with the domestic business environment. The results in Table 3 suggest that a small company has strategic advantages in the flexibility of its internationalization, which is in line with theoretical expectations.

**Table 3:** Probit model for foreign investment of Chinese small family business.

**Dependent Variable**	**Model 1**	**Model 2**	**Model 3**
	**International business expansion**		
Family Reputation (FR)		0.19^**^	0.06
		(2.27)	(0.49)
Family Involvement (FI)		−0.19^***^	−0.16^***^
		(−5.55)	(−3.89)
FR^*^ES			0.26^**^
			(2.50)
FI^*^ES			−0.16^***^
			(−3.50)
Entrepreneurial Spirit (ES)	0.25^***^	0.21^***^	0.10
	(3.93)	(3.31)	(1.44)
Domestic investment	0.07^***^	0.06^***^	0.07^***^
	(3.39)	(3.19)	(3.18)
Business environment	0.01	0.01	0.00
	(0.14)	(0.13)	(0.00)
Profit	0.04^**^	0.04^**^	0.04^*^
	(2.01)	(2.08)	(1.78)
Equity	−0.01	−0.02	−0.02
	(−0.59)	(−0.98)	(−0.77)
Diversification	0.05	0.03	0.05
	(0.58)	(0.27)	(0.56)
Firm size	−0.03	−0.07^*^	−0.06
	(−0.66)	(−1.65)	(−1.46)
Edu-higher school	−0.05	−0.06	−0.08
	(−0.37)	(−0.45)	(−0.53)
Edu-collage and above	0.10	0.01	−0.00
	(0.58)	(0.06)	(−0.02)
Region-central	−0.12	−0.15	−0.15
	(−1.13)	(−1.40)	(−1.33)
Region-western	−0.25^*^	−0.32^**^	−0.29^**^
	(−1.83)	(−2.37)	(−2.13)
Manu	−0.06	−0.01	0.00
	(−0.63)	(−0.15)	(0.02)
Constant	−2.16^***^	−1.34^***^	−1.35^***^
	(−6.19)	(−3.68)	(−3.62)
*N*	2,704	2,704	2,704
Prob. > chi2	0.000	0.000	0.000
R^2^	0.081	0.110	0.139

t statistics in parentheses. FR*ES, Family Reputation * Entrepreneurial Spirit; FI*ES, Family Involvement * Entrepreneurial Spirit.

* p < 0.1;

** p < 0.05;1

*** *p < 0.01*.

Model 2 provides the results for the main effect on IBE. The regression results show that family reputation has a significant and positive effect on IBE (coefficient = 0.19, *p* < 0.05). Outward foreign indirect investment in industrial fields is encouraged and supported by the Chinese government as a sign of growing Chinese market power, and substantial business expansion in international markets is welcomed by the local government and the public. It is acknowledged that only firms that perform well in the domestic market are in a position to implement a successful internationalization strategy, and foreign investment for business expansion enhances the business reputation and political assimilation of the owning family. A family business with a better family reputation (proxied by political status) will thus have greater incentives to expand its business internationally than a family business with a lower family reputation.

The results of Model 2 also suggest that family involvement has a significant and negative effect on IBE (coefficient = −0.19, *p* < 0.01). The SEWs for the family involvement of the owning family are based on family cohesion and embeddedness in the local social network. The internationalization process is always accompanied by a partial loss of family control associated with the need to leverage external resources and talents. A family business with a high level of family involvement will try to avoid foreign investment that involves a heavy commitment of resources and will accord a higher priority to risk-aversion and SEW (Choi et al., 2015). Thus, Hypothesis 1 (that family reputation is positively related to IBE) and Hypothesis 2 (that family involvement is negatively related to IBE) are supported by the results of this study.

Model 3 shows the results for the moderating effects of entrepreneurial spirit. The interaction term of family reputation × entrepreneurial spirit, which was added in Model 3, is positive and significant (coefficient = 0.26, *p* < 0.05). The results for the interaction effect show that the level of entrepreneurial spirit positively moderates the positive incentive of family reputation on IBE. In other words, a family business with a strong entrepreneurial spirit will have more incentive through family reputation to undertake IBE. Hypothesis 3 (that there is a moderating effect of entrepreneurial spirit on the relationship between family reputation and IBE) is thus supported.

An interaction term of family involvement × entrepreneurial spirit, added in Model 3, is found to have a significant and negative association with IBE (coefficient = −0.16, *p* < 0.01). Thus, an absence of entrepreneurial spirit will place higher importance on the preservation of family capital utilizing an international asset portfolio, which reflects a high level of family involvement. In the social context of the first generation of Chinese entrepreneurs following the opening up and reform of China, higher family involvement implies higher social embeddedness in the local community, which in the presence of strong entrepreneurial spirit places a higher priority on domestic investment than on foreign investment. In other words, the presence or absence of entrepreneurial spirit will make no substantial difference to the IBE for a family business with high family involvement. Hypothesis 4 (that there is a moderating effect of entrepreneurial spirit on the relationship between family involvement and IBE) is thus supported.

## Robustness Tests

To address the potential issues of self-selection and endogeneity, we use propensity score matching (PSM) analysis to check our results. The PSM analysis shows a good level of robustness of the empirical results even after controlling for the self-selection problem for both family reputation and family involvement (see below for details). Tobit models are also used to check the robustness of the empirical results. As shown below, the results of the Tobit models are similar to those of the Probit models, with the same significance. Accordingly, our results can be considered robust.

### Propensity Score Matching (PSM) Analysis

This study aims to identify the causal effect of family reputation and family involvement on the foreign investment of small family businesses. Unlike experimental studies in which sample cases are assigned at random to treatment or control conditions, any sample case can be subject to only one of the potential states of family reputation/family involvement, and we can only observe the outcome (IBE) in the treatment state or control state. Extending causal inference into observational studies is problematic since it is impossible to assign sample cases to treatment and control conditions (Morgan and Winship, 2007; Rosenbaum, 2010). Family businesses with positive reputations may be quite different from other family businesses in terms of company size, financial strength, diversification, perception of the business environment, and attitude to internationalization. Similarly, family businesses with lower family involvement may perform better in relation to corporate governance and management philosophy, which are not easily observed and are also associated with company size, financial strength, diversification, perception of the business environment, and attitude to internationalization. Therefore, any causal inference from the results in this study may be affected by selection bias.

To address endogeneity problems, this study used PSM to control for selection bias and to test the reliability of the regression results (see Table 3). PSM has major advantages over regression analysis, as matching on the propensity score can determine the distribution of covariates across sample cases exposed to the treatment and control conditions, as well as identifying sample cases that are comparable considering processes of self-selection based on previous empirical knowledge and theory (Morgan and Harding, 2006; Waibel et al., 2018). Thus, matching the propensity score is justified by the common support assumption and has to overlap the comparison groups of the analysis. PSM can be applied when the functional form of the relationship is not linear, as this method makes less stringent parametric assumptions.

For PSM purposes, this study constructs a dummy for family reputation, assigning 1 to a family business with political status in the CPC or CPPCC and 0 otherwise. A further dummy for family involvement assigns 1 to a family business with a level of family involvement greater than the median value in the sample and 0 otherwise. Using logistic models for PSM estimations, this study estimates propensity to family reputation and family involvement, respectively, including covariates. The group-specific propensity scores between family reputation groups and family involvement groups in both estimations show considerable overlap, allowing straightforward estimation of the treatment effect with comprehensive common support. The balancing assumption in both PSM estimations is also well-satisfied, with statistical comparisons across the treatment and control groups before and after the matchings indicating that both matchings are effective in balancing all variables affecting selection into family reputation/family involvement.

In the PSM estimation, the average treatment effect (ATT) of family reputation on IBE is positive and significant (coefficient = 0.0362, *p* < 0.05). As shown in Table 4, the difference between the treatment and control group drops from 0.0468 in the unmatched samples to 0.0362 in the matched samples and remains significant after controlling for selection bias. In the PSM estimation, the ATT of family involvement on IBE is negative and significant (coefficient = −0.0384, *p* < 0.01). Differences between the treatment and control groups in both matched and unmatched samples are negative and significant, implying that family involvement has a negative effect on IBE even after controlling for selection bias.

**Table 4:** PSM estimates of effect of family reputation/family involvement on international business expansion.

**Treatment variable**	**Sample**	**Treated**	**Controls**	**Difference**	**S.E**.	**T-stat**
Family reputation	Unmatched	0.0874	0.0406	0.0468	0.0099	4.73^***^
	ATT	0.0874	0.0512	0.0362	0.0166	1.97^**^
Family involvement	Unmatched	0.0291	0.0861	−0.0570	0.0086	−6.59^***^
	ATT	0.0291	0.0675	−0.0384	0.0118	−2.82^***^

*ATT, average treatment effect. BS = 1,000*.

** p < 0.05;

*** *p < 0.01*.

The results of the PSM estimations are substantially in line with those of the Probit model, and the hypotheses are supported in both cases (compare Tables 3, 4). This indicates that the results of this study are robust even after considering selection bias and the relevant endogeneity issues.

### Tobit Model Regression Analysis

This study uses Tobit models to check the robustness of its empirical results. The questionnaire includes items relating to the magnitude and purpose of investment in foreign countries in 2015, and Table 5 gives the results of the Tobit models. Comparison of Tables 3, 5 shows that the effects of family reputation and family involvement on IBE are supported by both the Probit and Tobit models. Likewise, the moderating effect of entrepreneurial spirit on the relationship between family reputation and IBE is robust in both cases. Moreover, the moderating effect of entrepreneurial spirit on the relationship between family involvement and IBE is supported in the Tobit model with statistical significance. Thus, a comparison of the Probit models, PSM analysis, and Tobit models confirm the robustness of the empirical results.

**Table 5:** The results of tobit models.

	**International business expansion**
Family reputation	2.25
	(1.54)
Family involvement	−1.98^***^
	(−4.64)
FR*ES	8.47^***^
	(3.49)
FI*ES	−2.99^***^
	(−3.96)
Profit	0.84^***^
	(3.83)
Equity	−0.11
	(−0.51)
Business environment	1.03
	(1.50)
Domestic investment	0.14
	(0.81)
Entrepreneurial spirit	3.11^***^
	(4.07)
Diversification	2.35^**^
	(1.97)
Firm size	0.21
	(0.50)
Edu-higher School	−2.37
	(−1.46)
Edu-collage and above	−1.34
	(−0.47)
Region-central	−0.12
	(−0.12)
Region-western	−1.20
	(−1.05)
Manu	2.00^**^
	(1.99)
Constant	−2.56
	(−0.76)
*N*	2,704
R^2^	0.016

t statistics in parentheses. FR*ES, Family Reputation * Entrepreneurial Spirit; FI*ES, Family Involvement * Entrepreneurial Spirit.

** p < 0.05;

*** *p < 0.01*.

## Discussion

This study proposes that the SEWs of an owning family will affect its attitude and preference in relation to IBE. It contributes to future research by distinguishing the effects of external and internal SEW on outward foreign investment. Research on outward foreign investment, a deep internationalization process with heavy resource commitment, offers a good context for exploring the functions and effects of dimensions of SEW on internationalization.

This study finds two relatively independent logics for external SEW and internal SEW in determining whether a family business will decide to invest abroad for the purposes of business expansion. Under an authoritarian regime in an emerging economy, a business family will usually seek protection in patron–client relationships. The importance of political connections to a family business has been extensively examined (Faccio, 2006; Berkman et al., 2010; Wu et al., 2018), and the political position of a business family is recognized as a vital facet of its social status. The reputations of business families are largely reflected in, and determined by, the official political titles and chairs in federations of industry and commerce held by family members or their agents. In China, only the most successful and prestigious businesspeople will have the opportunity to be assimilated politically by the local or national political system and chamber of commerce. A business family with a member in the CPC, CPPCC, or LFIC usually enjoys a more positive social reputation than others, and social reputation (especially political assimilation) is based on long-term business performance. Successful business expansion and interaction with local government economic strategy have always played an important role in building a family's reputation. In this way, business families with positive social reputations (proxied by political titles) will differ from other business families in their strategic choices for foreign investment. Successful IBE is a sign of powerful market competitiveness and prestigious market and social image, and an impetus to further enhance the family reputation, especially when local governments are keen to support firms to expand internationally. The preservation and improvement of external SEW based on family reputation offers extra motivation for the international expansion of a family business. In our empirical results, family reputation is significantly and positively associated with IBE.

On the other hand, family involvement, conceptualized as the inclination of family members to participate in the business operations of the family firm, is negatively associated with IBE. It is acknowledged that a family business with a high level of family involvement will assign a higher priority than other businesses to avoiding SEW risk (e.g., Choi et al., 2015). Foreign investment for business expansion usually involves a heavy commitment of resources (e.g., Gomez-Mejia et al., 2010) and leads to some loss of family control, which presents a threat to the preservation of SEW (Berrone et al., 2012; Zellweger et al., 2012). It is reasonable for a family business with a strong inclination toward family involvement to place a high priority on risk-averse investment for the sake of family continuity. The empirical results indicate that the effects of family involvement on IBE may differ from those of family reputation.

A SEW perspective is useful in explaining the strategic choices of Chinese family businesses concerning IBE. This study contributes to the literature by classifying distinct effects of external SEW (family reputation) and internal SEW (family involvement) and finds that the orders of priority proposed by external and internal SEW may be quite different. In the context of this study, family reputation places greater emphasis than family involvement on ambitious IBE. More importantly, the effects of family reputation and family involvement are moderated by entrepreneurial spirit. In a family business with high levels of entrepreneurial spirit, the owning family may focus on the preservation of external SEW, which will enhance the effect of family reputation on IBE. In a family business without high levels of entrepreneurial spirit, the owning family may focus instead on the preservation of internal SEW, which will have quite different effects. This study proposes that whether a family business chooses international expansion should be explained in terms of the distinction between external and internal SEW, with careful consideration as to which logic will dominate in a specific diversified business context.

This study provides insights into other factors in the choice of IBE. The results in Table 3 indicate that profit has a positive and significant effect on IBE; that is, profitability is a basic factor in motivating the internationalization of a family business. Similarly, the domestic investment of a family business is positively and significantly associated with IBE. Location in the western region of China is negatively and significantly connected with IBE.

## Conclusion and Limitations

The objective of this study was to analyze the effect of multiple dimensions of SEW on IBE. In a sample of 2,704 small family businesses in China, family reputation (i.e., external SEW) had a positive effect on IBE, while family involvement (i.e., internal SEW) had a negative effect. This study also argues that entrepreneurial spirit may change the owning family's priorities in pursuit of SEW, moderating the effect of family reputation and family involvement on IBE. In this sample, entrepreneurial spirit reinforces the positive effect of family reputation on IBE and enhances the negative relationship between family involvement and IBE.

This study advances our understanding of the international expansion of family businesses from the SEW perspective in four key ways, theoretically and empirically. First, by classifying SEW into external sources proxied by family reputation and internal sources proxied by family involvement, our study sheds light on the multiple dimensions of SEW and their effects on IBE, thereby contributing to the literature on SEW (Debicki et al., Gómez-Mejía et al., 2007; Berrone et al., 2012; Sciascia et al., 2014; Cesinger et al., 2016; Hauck et al., 2016; Yang et al., 2018). Our findings are in line with studies that have argued for the importance of the heterogenicity of a family firm (Stanley et al., 2017; Yang et al., 2018). Family heterogenicity in the pursuit of SEW and its multiple dimensions have been ignored in the literature to date (Debicki et al., 2016; Hauck et al., 2016; Yang et al., 2018), and the concept of SEW has been indiscriminately treated and measured by proxies such as family ownership and family control (Gómez-Mejía et al., 2007; Berrone et al., 2012; Sciascia et al., 2014; Cardella et al., 2020). Our study finds heterogenic effects of external and internal SEW on the international expansion of family businesses, thus supporting the idea of heterogenicity in a family business's pursuit of SEW (Gómez-Mejía et al., 2007; Gomez-Mejia et al., 2011; Chirico and Nordqvist, 2010; Chrisman et al., 2012). In this way, our study contributes to the exploration of the black box of SEW, offering empirical evidence in the context of the foreign investment of small family businesses in China.

Second, this study explored the moderating effect of entrepreneurial spirit on the relationship between SEWs and IBE. In previous studies, entrepreneurial spirit is usually missing from the discussion of the SEW of family businesses (Gómez-Mejía et al., 2007; Berrone et al., 2012) and the influence of SEW on entrepreneurial orientation (Hernández-Perlines et al., 2019). Our study finds that the order of priority accorded to SEW in a family business varies with the presence or absence of entrepreneurial spirit. Unlike previous analyses of how entrepreneurial orientation works directly in the internationalization of family business (Claver et al., 2007; Arregle et al., 2012), our study explores the moderating effect of entrepreneurial spirit, which has received much less attention in the literature. It enriches the moderating conditions of the linkage of SEW and internationalization, building on previous explorations of the moderating effects of founder CEO and family succession intention (Yang et al., 2018), business context (Naldi et al., 2013), corporate governance (Lu et al., 2015), and other variables. In this way, our study contributes to the SEW perspective on family business internationalization theory.

Third, the study investigated IBE in terms of foreign investment rather than factors such as export intensity and export propensity (Ganotakis and Love, 2012). Foreign investment usually involves relatively heavy commitment of resources and thus offers an excellent context in which to explore the functions and effects of SEW on internationalization (Gomez-Mejia et al., 2010; Kumpikait-Valiunien et al., 2021). The foreign investment of family businesses in emerging markets is itself an interesting topic, and this study offers a useful perspective for understanding the decision-making processes of owning families, enriching the literature on their motives for international expansion by showing that they are heterogenetic and context-dependent from the SEW perspective.

Fourth, this study offers insights into the failure and resilience of international expansion, which for most owning families is a form of entrepreneurship (Oviatt and McDougall, 2005). Our results show to some extent that SEW is an important psychology-related factor in the decisions of family businesses on international expansion; from this, it is reasonable to infer that SEW is also an important psychology-related factor in the persistence of international expansion in family businesses. Internal SEW worries, which originate for the most part in the psychological processes of family members, may hinder the family business's strategy for international expansion, leading to stagnation of its entrepreneurship. In contrast, external SEW may inspire family businesses to increase the resilience of the company in a new phase of international entrepreneurship, and that resilience can in turn enhance the effect of external SEW on further international expansion.

Concerning the practical implications of our findings, we first suggest that a family business that intends to invest abroad should manage its SEW, giving serious consideration to how to leverage the pursuit of external SEW and how to address the concerns of family members about internal SEW. Second, we suggest that the entrepreneurship orientation should be enhanced if the owning family business intends to pursue internationalization partly for the sake of external SEW. Full recognition of the competitive advantages offered by IBE will help to relieve the negative effects of internal SEW worries, making the entrepreneurship orientation more helpful in encouraging international investment. Third, local government can play an important role in the encouragement of business expansion by giving higher social appraisal to international investment when internationalization-orientated policy encourages family businesses to invest abroad.

This study is not without limitations. First, given the complexity of the internationalization of family businesses and the multiple dimensions of SEW, it is exploratory in nature and proxies external and internal SEW by family reputation and family involvement, respectively. In reality, the meaning and constituent elements of external and internal SEW go far beyond family reputation and family involvement. More effort is therefore required to shed light on the black box of SEWs and their effects on the internationalization of family businesses, including mediating and moderating effects. Second, this study focuses on a single country (China), and the results might reflect some peculiarities of the national economic and social context. Our findings are very specific to the Chinese transitional context, and caution should be exercised in an attempt to generalize to family businesses in other countries. Third, although it would be interesting to explore the issues raised here using a cross-country dataset, we lacked samples from other countries. The family context is, to some extent, country-specific; family businesses in America and Western Europe may be quite different from family businesses in East Asia. We recommend that future studies undertake a cross-country comparison to detect the similarities and differences among family businesses in a range of cultural and economic contexts. Fourth, our study assumes that intra-heterogeneity among Chinese family businesses is relatively small. However, this assumption may underestimate the wide variations found among local cultures, industries, succession generations, and foundation background. Therefore, exploration of intra-China heterogeneity on this issue is a task for future research.

## Data Availability Statement

The raw data supporting the conclusions of this article will be made available by the authors, without undue reservation.

## Ethics Statement

Ethical review and approval were not required for the study on human participants in accordance with local legislation and institutional requirements. The patients/participants provided written informed consent to participate in this study.

## Author Contributions

All authors listed have made a substantial, direct and intellectual contribution to the work, and approved it for publication.

## Conflict of Interest

The authors declare that the research was conducted in the absence of any commercial or financial relationships that could be construed as a potential conflict of interest.
